# Long-term effect of the German Parkinson’s disease multimodal complex treatment: a clinical trial with randomized allocation

**DOI:** 10.1186/s12883-026-05039-5

**Published:** 2026-06-04

**Authors:** Konstantin G. Heimrich, Aline Schönenberg, Sarah Mendorf, Hannah M. Mühlhammer, Ulrike Teschner, Thomas Lehmann, Stefan Brodoehl, Tino Prell

**Affiliations:** 1 Department of Geriatrics, Jena University Hospital, Am Klinikum 1, Jena, 07747 Germany; 2 Department of Neurology, Jena University Hospital, Jena, Germany; 3 Department of Geriatrics, Halle University Hospital, Halle, Germany; 4 Center for Clinical Studies, Jena University Hospital, Jena, Germany

**Keywords:** Parkinson´s disease, Multidisciplinary care, Depression, Quality of life

## Abstract

**Background:**

The German Parkinson´s disease multimodal complex treatment (PD-MCT) is a structured multidisciplinary inpatient treatment for people with Parkinson’s disease (PwPD). However, data on its long-term effect are limited.

**Methods:**

A monocentric, non-blinded, clinical trial with randomized allocation and six-month follow-up was conducted at the Department of Neurology of the Jena University Hospital. At total of 120 patients admitted for PD-MCT were allocated 1:1 to two predefined treatment durations (short: 7–13 days; long: 14–20 days) according to the German Operation and Procedure Classification System (OPS; code 8-97d). The primary outcome was change in the Movement Disorder Society sponsored revision of the unified Parkinson’s disease rating scale (MDS-UPDRS) part II after six months. Secondary outcomes included change in quality of life assessed by the Parkinson’s Disease Questionnaire 8 (PDQ-8) and the influence of treatment duration. Analyses were performed as-treated.

**Results:**

In 93 PwPD, MDS-UPDRS II improved by 3 points (*r* = 0.435; *p* < 0.001), with 52.7% achieving a clinically relevant improvement. Improvement was associated with higher baseline MDS-UPDRS II (odds ratio [OR] 1.16, 95% confidence interval [CI] 1.07–1.27; *p* < 0.001), lower Hoehn and Yahr stage (OR 0.29, 95% CI 0.09–0.97; *p* = 0.045), and fewer nonmotor symptoms (OR 0.88, 95% CI 0.77-1.00; *p* = 0.042) (*x*^2^(3) = 14.94; *p* = 0.002; *R*^2^ = 0.22). PDQ-8 improved by 8 points (*r* = 0.524; *p* < 0.001), with 58.1% showing clinically relevant improvement, which was associated with lower Hoehn and Yahr stage (OR 0.30, 95% CI 0.10–0.92; *p* = 0.035), and more depressive symptoms (OR 1.17, 95% CI 1.04–1.33; *p* = 0.012) (*x*^2^(2) = 10.53; *p* = 0.005; *R*^2^ = 0.15). Treatment duration had no significant influence on MDS-UPDRS II (*p* = 0.611, Eta² = 0.001) or PDQ-8 (*p* = 0.809, Eta² = 0.001).

**Conclusions:**

PD-MCT is an effective multidisciplinary inpatient treatment with clinically relevant long-term effects up to six months. In clinical practice, the shorter treatment duration may be similarly effective in selected patients.

**Trial registration:**

German Clinical Trials Register, DRKS00025225, registered 30 June 2021, https://drks.de/search/de/trial/DRKS00025225.

**Supplementary Information:**

The online version contains supplementary material available at 10.1186/s12883-026-05039-5.

## Background

Parkinson´s disease (PD) is one of the most common neurodegenerative disorders with motor and various nonmotor symptoms [1], resulting in severe disability and impacting patients’ quality of life [2–6]. With the progressive deterioration of motor and nonmotor functions, the individual medical treatment has to be adapted continuously [7]. Due to the complexity of symptoms, a multidisciplinary approach to treatment is necessary to preserve patients’ health. Consequently, there has been a growing implementation of multidisciplinary inpatient PD treatment approaches [8]. In Germany, this approach is known as Parkinson’s disease Multimodal Complex Treatment (PD-MCT) [9], which is integrated into the German health insurance system and conducted in line with the specifications of the Operation and Procedure Classification System (OPS), the national coding system for medical procedures. Accordingly, there are precise specifications for carrying out PD-MCT, ensuring comparability of the program across hospitals. The duration of PD-MCT ranges from a minimum of 7 days to more than 21 days, thus PD-MCT can be differentiated into a short treatment over a period of 7–13 days (OPS 8-97d.0), a long treatment of 14–20 days (OPS 8-97d.1), and a treatment ≥ 21 days (OPS 8-97d.2).

In Germany, the number of PD-MCT procedures has been rising, with 15,303 treatments conducted across hospitals in 2022 [10]. However, despite its wide implementation, there is limited data on its effectiveness. To the best of our knowledge only four studies assessed the effect of PD-MCT [11–15]. One study on 126 people with PD (PwPD) found that a treatment regimen of approximately three weeks led to improvements in both motor and nonmotor scales [11]. Another prospective non-randomized observational study involving 47 PwPD indicated that PD-MCT was effective in alleviating motor symptoms, depression, and health-related quality of life (HRQoL) for up to six weeks [12, 13]. Two observational retrospective analyses confirmed the positive effects of PD-MCT [14, 15]. Of them, one study on 591 PwPD with a follow-up period of four weeks revealed that approximately 48% of PwPD exhibited a clinically relevant improvement in their motor experiences of daily living at follow-up [15].

Regarding treatment duration, only a small percentage of PwPD is treated for less than 14 days in the PD-MCT [9]. However, there are insufficient data regarding the effectiveness of the more frequently conducted longer treatment duration in comparison to the shorter treatment duration. Only one of the above-mentioned studies presented data regarding the shorter treatment duration of PD-MCT [14], demonstrating that the shorter PD-MCT is an efficacious treatment option in certain cases. However, in order to assess the effectiveness of both versions of the PD-MCT in comparison, a prospective study setting with randomization into the two treatment durations is required. This data is highly valuable as evidence of the effectiveness of the short PD-MCT would enable a redirection of resources, enabling more PwPD to receive highly specialized treatment.

In summary, previous research indicates that PD-MCT is an effective treatment approach, with a positive effect lasting at least four weeks. However, two important questions remain: First, how long does the positive treatment effect last? Second, does the treatment duration have a significant impact on its effectiveness? Accordingly, the primary objective of the trial was to reveal whether PD-MCT has a long-term effect six months after discharge regarding motor experiences of daily living. The secondary objectives were to determine the long-term effect regarding HRQoL, and to determine whether the treatment duration (short vs. long) has a significant influence on its long-term effect. Given the increasing prevalence of PD and the associated challenges to the healthcare system, this information is of great importance.

## Methods

### Study design and setting

We conducted a monocentric prospective clinical trial with randomized allocation to the two most relevant and predefined routine care categories of PD-MCT with six-month follow-up at the Department of Neurology of the University Hospital Jena, Germany. The study is registered at the German Clinical Trials Register (registration number: DRKS00025225). Inclusion criteria were the presence of idiopathic PD according to the Movement Disorder Society (MDS) criteria [16], and admission for PD-MCT. Exclusion criteria were the inability to give informed consent to participate in the study, and severe cognitive impairment with the inability to complete a questionnaire. Written informed consent was obtained from each patient.

### Study population, stratification and randomization

Between July 2021 and March 2023, PwPD admitted to the neurology ward for PD-MCT were screened and randomly allocated 1:1 to short (7–13 days; OPS 8-97d.0) or long (14–20 days; OPS 8-97d.1) PD-MCT and stratified by sex using a computerized randomization tool from the Jena University Hospital Center for Clinical Studies (PaRANDies). Randomized allocation was used to reduce selection bias at admission. However, for ethical and medical reasons, the treatment duration could subsequently be adjusted if clinically necessary to avoid any harm.

### Intervention

After allocation, PwPD received PD-MCT according to the specifications of the OPS 8-97d. PD-MCT is a structured inpatient multidisciplinary treatment program that is standardized across German hospitals by OPS requirements, including a predefined treatment duration, a minimum treatment intensity of at least 7.5 h weekly, and involvement of multiple therapeutic disciplines. Thus, PD-MCT included a neurologist-led evaluation and optimization of antiparkinsonian medications, specialized nursing care, and personalized non-pharmacological therapies. These consisted of physiotherapy, occupational therapy, speech and language therapy, and, when necessary, psychological or neuropsychological support, as well as social work interventions.

The therapeutic concept was the same for both groups. The only difference between the two groups was the duration of the treatment.

### Primary outcome

The primary outcome was to determine the long-term effect of PD-MCT six months after discharge as a change in motor experiences of daily living using the Movement Disorder Society sponsored revision of the unified Parkinson’s disease rating scale part II (MDS-UPDRS II) [17]. After study registration, we changed the primary outcome from MDS UPDRS III to MDS UPDRS II to enable telephone follow-up during the COVID-19 pandemic.

### Secondary outcomes

Secondary outcomes were 1) to determine a change in HRQoL using the PD questionnaire (PDQ-8) [18, 19] at follow-up, and 2) to assess the influence of treatment duration (short vs. long) on the long-term effect of PD-MCT regarding both MDS-UPDRS II and PDQ-8 (as-treated analysis).

### Covariates

Based on the previous literature, several additional variables were selected as covariates, including *age* (metric), *sex* (male “0”, female “1”), and PD-specific parameters such as *Hoehn and Yahr* stage (ordinal) [20], motor impairment according to the MDS-UPDRS part II and III (*MDS-UPDRS II* and *III*, metric) [17] in the medication ON state, levodopa equivalent daily dose (*LEDD* [mg], metric) [21], the number of non-motor symptoms as assessed by the non-motor symptoms questionnaire (*NMSQ*, metric) [22], cognition (Montreal Cognitive Assessment, *MoCA*, metric) [23], and depressive symptoms (Patient Health Questionnaire-9, *PHQ-9*, metric) [24]. In addition, a performance-oriented mobility assessment was performed (*Tinetti* test, metric) [25]. These covariates were determined at admission to PD-MCT (baseline). Moreover, *MDS-UPDRS III*, *LEDD*, and *Tinetti* were again tested at discharge from PD-MCT.

### Sample size calculation

The primary aim of the study was to detect a minimal clinically relevant improvement of the MDS-UPDRS II between baseline and six months after discharge. This improvement was set at 3 points [26]. With a significance level of *p* < 0.05 (two-tailed) and a power level of 80%, assuming a standard deviation (SD) of differences of 8 points, a minimum of 58 pairs were required. For the secondary outcome of HRQoL and its long-term effect, a post-hoc sample size calculation at a significance level of *p* < 0.05 revealed that the sample of 93 PwPD at follow-up has a power of 99.9% to detect the mean difference for PDQ-8 of 15.5 ± 8.1.

### Statistical analyses

For statistical analyses, IBM SPSS version 29, R version 4.3.0, and jamovi version 2.2 were used. Statistical significance for all tests was set at *p* < 0.05. The primary and secondary outcomes were tested *as-treated*, because treatment duration adjustments were permitted for ethical and clinical reasons.

Data were checked for normality using the Shapiro-Wilk test. Continuous variables with normal distribution are presented as mean ± standard deviation (SD), whereas non-normally distributed variables are presented as median and interquartile range (IQR). Categorical variables are presented as absolute numbers and percentages. Group comparisons were performed using independent-samples t-tests or paired t-tests for normally distributed data, and Mann–Whitney U tests or Wilcoxon signed-rank tests for non-normally distributed data. Effect sizes are reported as Cohen’s d for normally distributed data (> 0.2: low; > 0.5: moderate; > 0.8: strong), or rank biserial correlation for non-normally distributed data (> 0.1: low; > 0.3: moderate; > 0.5: strong) [27].

Binominal logistic regression with backward selection (likelihood ratio) were performed to identify predictors of clinically relevant improvement (MDS-UPDRS II; PDQ-8). PwPD were lost to follow-up if unreachable by telephone after five attempts. Responders showed an improvement of at least 3 points in the MDS-UPDRS II [26] or 6 points in the PDQ-8 [28], corresponding to the minimal clinically important differences (MCID). Autocorrelation and multicollinearity were excluded (|r| < 0.8). Linearity was assessed using the Box–Tidwell procedure. Outliers (SD of the studentized residuals > 3; leverages > 0.2) were subsequently excluded. Baseline MDS-UPDRS II was included as a marker of initial disease severity; however, this requires cautious interpretation due to its relationship with change scores.

To assess the influence of the treatment duration, mixed Type III ANOVAs were performed with timepoint (baseline/follow up) as within-subject factor and treatment duration (short/long) as between-subject factor using the the R-Packages *rstatix*, *afex* and *performance*.

## Results

### Participants

**Fig. 1:**
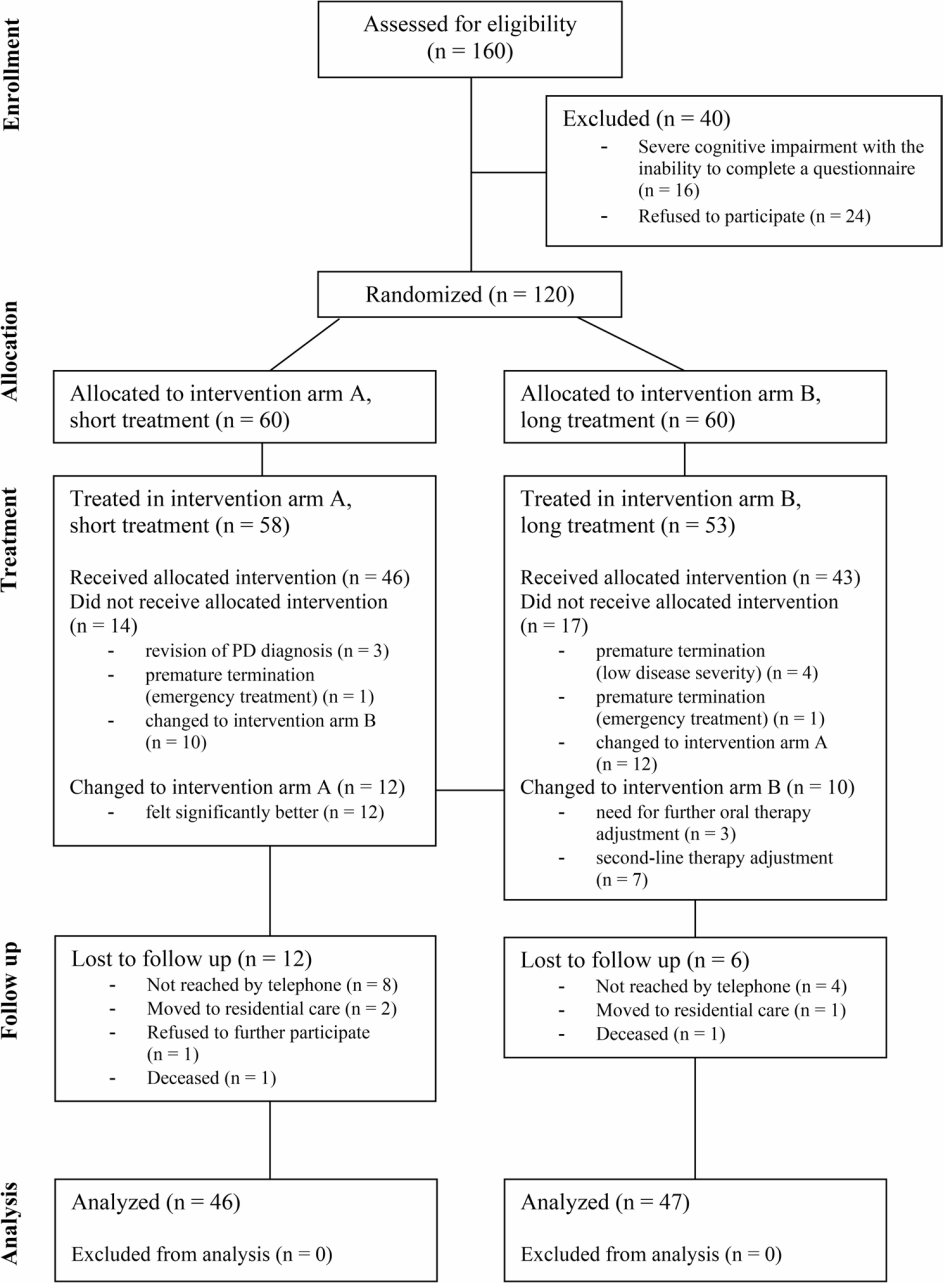
CONSORT diagram showing the flow of participants. Intervention arm A: participants were treated for 7–13 days within PD-MCT. Intervention arm B: participants were treated for 14–20 days within PD-MCT. n: Number of people. PD: Parkinson’s disease

Figure 1 is showing the flow of PwPD through each stage of the trial. Between July 2021 and March 2023, a total of 160 PwPD were admitted for PD-MCT. Of those, 120 (75%) were randomized. Of 120 randomized PwPD, nine did not complete PD-MCT and were therefore excluded. Six months after discharge a telephone follow-up was conducted in 93 PwPD to assess the long-term effect of PD-MCT (OPS 8-97d.0, short treatment, *n* = 46; OPS 8-97d.1, long treatment, *n* = 47). The last follow-up was carried out in September 2023. Of the initial 111 PwPD, 18 were lost to follow-up.

### Baseline characteristics and changes within the PD-MCT

Table 1 displays the characteristics of the initial 111 PwPD included in the study. They had a median age of 72 years (IQR 66–78). Upon admission, most PwPD had postural instability (Hoehn and Yahr stage ≥ 3) and moderate motor impairment according to a mean score of 39.5 ± 13.1 points on the MDS-UPDRS III. The median LEDD was 690 mg (IQR 590–1136). On average, PwPD reported 11.5 ± 4.9 non-motor symptoms according to the NMSQ. The MoCA revealed that 8.4% of PwPD had normal cognition, 43.0% had mild cognitive impairment (MoCA range 21 to 25 points), and 48.6% had PD dementia [23].

**Table 1:** Characteristics of the baseline study population (*n* = 111)

	Study population
	(*n* = 111)
Age, years [median; IQR]	72 (66–78)
Sex, female [*n*; %]	46 (41.4)
Hoehn and Yahr, stage [median; IQR]	3 (3–3)
NMSQ, points [mean ± SD]	11.5 ± 4.9
PHQ-9, points [mean ± SD]	7.9 ± 4.3
MoCA, points [median; IQR]	21 (18–23)
MDS-UPDRS II, points [median; IQR]	18 (12–25)
MDS-UPDRS III admission, points [mean ± SD]	39.5 ± 13.1
LEDD admission, mg [median; IQR]	690 (500–1136)
Tinetti admission, points [median; IQR]	21 (14–26)

Values are given as mean and standard deviation (SD) for normally distributed data or as median and interquartile range (IQR) for non-normally distributed data; categorial parameters are given as absolute values and percentages

*LEDD *levodopa equivalent daily dose, *MDS-UPDRS *Movement Disorder Society sponsored revision of the unified Parkinson’s disease rating scale, *MoCA *Montreal Cognitive Assessment, *NMSQ *Non-Motor Symptoms Questionnaire, *PHQ-9 *Patient Health Questionnaire-9

Subgroup analysis revealed that patients receiving short-term treatment had better mobility (Tinetti: *p* = 0.003; *r* = 0.326), and lower disease severity regarding Hoehn and Yahr stage (*p* = 0.038; *r* = 0.187), MDS-UPDRS II (*p* = 0.008; *r* = 0.293), and LEDD at admission (*p* = 0.031; *r* = 0.239) (see Table 2). After receiving treatment within the PD-MCT, PwPD demonstrated improvements in motor function (MDS-UPDRS III), increased LEDD, and enhanced motor performance according to the Tinetti score. These improvements were consistent regardless of the duration of PD-MCT treatment duration (Table 2). On average, motor function improved by 13.0 ± 8.8 points, however, three PwPD experienced a worsening of MDS-UPDRS III scores. Median LEDD increase was 170 mg (IQR 13–375), with an increase in LEDD required in 84 (75.7%) and a decreased in 26 (23.4%) PwPD. Functional motor performance as measured by the Tinetti test improved by 3 points (IQR 1–5), while five PwPD (4.6%) demonstrated a worsening of Tinetti scores over the course of their hospital stay.

**Table 2:** Baseline characteristics and treatment changes of the short and long treatment group

	Short treatment	Long treatment	df	*p*	*r*
	(*n* = 58)	(*n* = 53)			
Age, years [median; IQR]	72 (65–78)	72 (67–78)	109	0.531	/
Sex, female [*n*; %]	23 (39.7)	23 (43.4)	109	0.693	/
Hoehn and Yahr, stage [median; IQR]	3 (2.5–3)	3 (3–3)	109	0.038	0.187
NMSQ, points [mean ± SD]	10.9 ± 4.7	12.1 ± 5.0	109	0.299	/
PHQ-9, points [mean ± SD]	7.7 ± 4.5	8.1 ± 4.1	107	0.685	/
MoCA, points [median; IQR]	21 (17–24)	20 (19–23)	105	0.913	/
MDS-UPDRS II, points [median; IQR]	14.5 (11–23)	21 (14–26.5)	109	0.008	0.293
MDS-UPDRS III admission, points [mean ± SD]	37.5 ± 11.9	41.7 ± 14.1	109	0.113	/
MDS-UPDRS III discharge, points [mean ± SD]	25.8 ± 11.7	27.1 ± 11.9	107	0.407	/
LEDD admission, mg [median; IQR]	601 (435–872)	745 (555–1322)	109	0.031	0.239
LEDD discharge, mg [median; IQR]	708 (575–1083)	940 (652–1724)	109	0.045	0.221
Tinetti admission, points [median; IQR]	23 (17–26)	17 (13–23)	108	0.003	0.326
Tinetti discharge, points [median; IQR]	26 (20–28)	23 (17–27)	107	0.029	0.242

Short treatment: PwPD who were treated for 7–13 days (OPS 8.97d.0). Long treatment: PwPD who were treated for 14–20 days (OPS 8.97d.1). Values are given as mean and standard deviation (SD) for normally distributed data or as median and interquartile range (IQR) for non-normally distributed data; categorial parameters are given as absolute values and percentages

Group comparisons of short and long treatment were performed using independent-samples t-tests for normally distributed data, and Mann–Whitney U tests for non-normally distributed data. Effect sizes are reported as Cohen’s d for normally distributed data or rank biserial correlation for non-normally distributed data

*LEDD *levodopa equivalent daily dose, *MDS-UPDRS *Movement Disorder Society sponsored revision of the unified Parkinson’s disease rating scale, *MoCA *Montreal Cognitive Assessment, *NMSQ *Non-Motor Symptoms Questionnaire, *PHQ-9 *Patient Health Questionnaire-9

### Primary outcome: MDS-UPDRS II after six months

The characteristics of the 93 PwPD included in the longitudinal analysis are presented in Table 3. The PwPD reported a median improvement of 3 points on the MDS-UPDRS II (IQR − 1–7)(*r* = 0.435; *p* < 0.001). Sensitivity analyses confirmed significant improvements in MDS-UPDRS II for PwPD receiving short and long PD-MCT treatments (short treatment: median improvement 2 points, IQR − 1–6.2, *r* = 0.423, *p* = 0.002; long treatment: median improvement 3 points, IQR − 2–7.25, *r* = 0.449, *p* = 0.010). Using the MCID, the MDS-UPDRS II improvement can be considered clinically relevant in 48 PwPD (52.7%) [26].

**Table 3:** Characteristics of the longitudinal study population (*n* = 93)

	Baseline	Follow-up	df	*p*	*r*
	(*n* = 93)	(*n* = 93)			
Age, years [median; IQR]	72 (66–77.5)	/			
Sex, female [*n*; %]	40 (43.0)	/			
Hoehn and Yahr, stage [median; IQR]	3 (3–3)	/			
NMSQ, points [mean ± SD]	10.9 ± 4.8	/			
PHQ-9, points [mean ± SD]	7.3 ± 4.1	/			
MoCA, points [median; IQR]	21 (18–24)	/			
MDS-UPDRS III admission, points [mean ± SD]	38.5 ± 12.5	/			
LEDD admission, mg [median; IQR]	645 (450–1130)	/			
Tinetti admission, points [median; IQR]	22 (16–26)	/			
MDS-UPDRS II, points [median; IQR]	16 (12–24)	14 (7.5–21)	90	< 0.001	0.435
PDQ-8 summary index, points [median; IQR]	31 (22–44)	25 (9–38)	92	< 0.001	0.524
PDQ-8, item 1: Mobility	2 (1–3)	2 (1–2)	92	0.291	/
PDQ-8, item2: Activities of daily living	1 (0–3)	1 (0–3)	92	0.562	/
PDQ-8, item 3: Emotional well-being	1 (1–2)	1 (0–2)	92	0.062	/
PDQ-8, item 4: Social support	1 (0–2)	0 (0–0)	91	< 0.001	0.727
PDQ-8, item 5: Cognition	2 (1–2)	1 (0–2)	92	0.007	0.375
PDQ-8, item 6: Communication	1 (0–2)	0 (0–1)	92	< 0.001	0.582
PDQ-8, item 7: Bodily discomfort	2 (1–3)	1 (0–2)	92	< 0.001	0.613
PDQ-8, item 8: Stigma	0 (0–2)	0 (0–1)	92	0.029	0.355

Values are given as mean and standard deviation (SD) for normally distributed data or as median and interquartile range (IQR) for non-normally distributed data; categorial parameters are given as absolute values and percentages. Group comparisons were conducted using paired t test for normally distributed differences and Wilcoxon signed rank test for non-normally distributed differences. Effect sizes were given as Cohen’s d for normally distributed data and Rank biserial correlation for non-normally distributed data

*LEDD *levodopa equivalent daily dose, *MDS-UPDRS *Movement Disorder Society sponsored revision of the unified Parkinson’s disease rating scale, *MoCA *Montreal Cognitive Assessment, *NMSQ *Non-Motor Symptoms Questionnaire, *PDQ-8 *Parkinson’s Disease Questionnaire-8, *PHQ-9 *Patient Health Questionnaire-9

Next, to understand the factors contributing to a clinically relevant improvement of the MDS-UPDRS II (responders), we performed logistic regression analyses. A relevant improvement of the MDS-UPDRS II was associated with higher MDS-UPDRS II (OR 1.16, 95% CI 1.07–1.27; *p* < 0.001), lower Hoehn and Yahr stage (OR 0.29, 95% CI 0.09–0.97; *p* = 0.045), and less nonmotor symptoms at admission (OR 0.88, 95% CI 0.77-1.00; *p* = 0.042) (*x*^2^(3) = 14.94; *p* = 0.002; Nagelkerke *R*^2^ = 0.22) (see Table 4). 

**Table 4:** Regression analysis, relevant improvement of the MDS-UPDRS II after six months

		B	SE	*p*	OR	CI lb	CI up
Step 1	Age	0.013	0.031	0.679	1.013	0.953	1.077
	Sex	-0.541	0.523	0.301	0.582	0.209	1.622
	Hoehn and Yahr	-0.806	0.706	0.253	0.447	0.112	1.781
	NMSQ	-0.160	0.092	0.083	0.852	0.711	1.021
	PHQ9	0.010	0.085	0.904	1.010	0.855	1.193
	MoCA	0.055	0.070	0.429	1.056	0.922	1.211
	MDS-UPDRS II	0.202	0.062	0.001	1.224	1.084	1.382
	MDS-UPDRS III admission	-0.021	0.028	0.458	0.979	0.926	1.035
	LEDD admission	0.000	0.001	0.385	1.000	0.998	1.001
	Tinetti admission	0.042	0.059	0.477	1.043	0.929	1.170
	Treatment duration	0.162	0.529	0.760	1.175	0.417	3.312
	Constant	-0.911	3.939	0.817	0.402		
Step 9	Hoehn and Yahr	-1.223	0.610	0.045	0.294	0.089	0.972
	NMSQ	-0.130	0.064	0.042	0.878	0.774	0.995
	MDS-UPDRS II	0.152	0.044	< 0.001	1.164	1.068	1.269
	Constant	2.450	1.572	0.119	11.583		

Values were obtained using binominal logistic regression analysis with backward selection (likelihood ratio) to identify predictors of clinically relevant improvement of the MDS-UPDRS II from admission to follow-up six months after PD-MCT. B: Unstandardized regression coefficient

*CI lb* Lower bound of the 95% confidence interval, *CI ub *upper bound of the 95% confidence interval, *LEDD *levodopa equivalent daily dose, *MDS-UPDRS *Movement Disorder Society sponsored revision of the unified Parkinson’s disease rating scale, *MoCA *Montreal Cognitive Assessment, *NMSQ *Non-Motor Symptoms Questionnaire, *OR *Odds Ratio, *PHQ-9 *Patient Health Questionnaire-9

### Secondary outcome: HRQoL after six months

There was an improvement of 8.1 ± 15.5 points in the PDQ-8 (*r* = 0.524; *p*< 0.001) after six months (Table 3). This improvement was observed in both the short (mean improvement 8.5 ± 15.8 points) and long PD-MCT (mean improvement 7.7 ± 15.2 points). Of 93 PwPD, 54 (58.1%) had a clinically relevant improvement of PDQ-8 [28]. In the logistic regression, this improvement was associated with lower Hoehn and Yahr stage (OR 0.30, 95% CI 0.10–0.92; *p* = 0.035), and more depressive symptoms at baseline (OR 1.17, 95% CI 1.04–1.33; *p* = 0.012) (*x*^2^(2) = 10.53; *p* = 0.005; Nagelkerke R^2^ = 0.15) (see Table 5).

**Table 5:** Regression analysis, relevant improvement of HRQoL after six months

		B	SE	*p*	OR	CI lb	CI up
Step 1	Age	0.025	0.031	0.419	1.025	0.965	1.089
	Sex	0.273	0.508	0.590	1.314	0.486	3.554
	Hoehn and Yahr	-1.535	0.764	0.044	0.215	0.048	0.963
	NMSQ	-0.060	0.085	0.481	0.942	0.796	1.113
	PHQ9	0.255	0.100	0.011	1.290	1.060	1.570
	MoCA	0.049	0.064	0.444	1.050	0.926	1.191
	MDS-UPDRS II	-0.030	0.047	0.530	0.971	0.885	1.065
	MDS-UPDRS III admission	0.000	0.027	0.996	1.000	0.948	1.055
	LEDD admission	0.000	0.001	0.553	1.000	0.999	1.001
	Tinetti admission	-0.093	0.059	0.119	0.912	0.812	1.024
	Treatment duration	-0.656	0.526	0.213	0.519	0.185	1.455
	Constant	3.702	3.996	0.354	40.533		
Step 10	Hoehn and Yahr	-1.205	0.570	0.035	0.300	0.098	0.916
	PHQ9	0.160	0.064	0.012	1.174	1.036	1.330
	Constant	2.660	1.610	0.099	14.297		

Values were obtained using binominal logistic regression with backward selection (likelihood ratio) to identify predictors of clinically relevant improvement of HRQoL (PDQ-8) from admission to follow-up six months after PD-MCT. B: Unstandardized regression coefficient

*CI lb *Lower bound of the 95% confidence interval, *CI ub *upper bound of the 95% confidence interval, *LEDD *levodopa equivalent daily dose, *MDS-UPDRS *Movement Disorder Society sponsored revision of the unified Parkinson’s disease rating scale, *MoCA *Montreal Cognitive Assessment, *NMSQ *Non-Motor Symptoms Questionnaire, *OR *Odds Ratio, *PHQ-9 *Patient Health Questionnaire-9

### Secondary outcome: influence of the treatment duration on the long-term effect

**Fig. 2:**
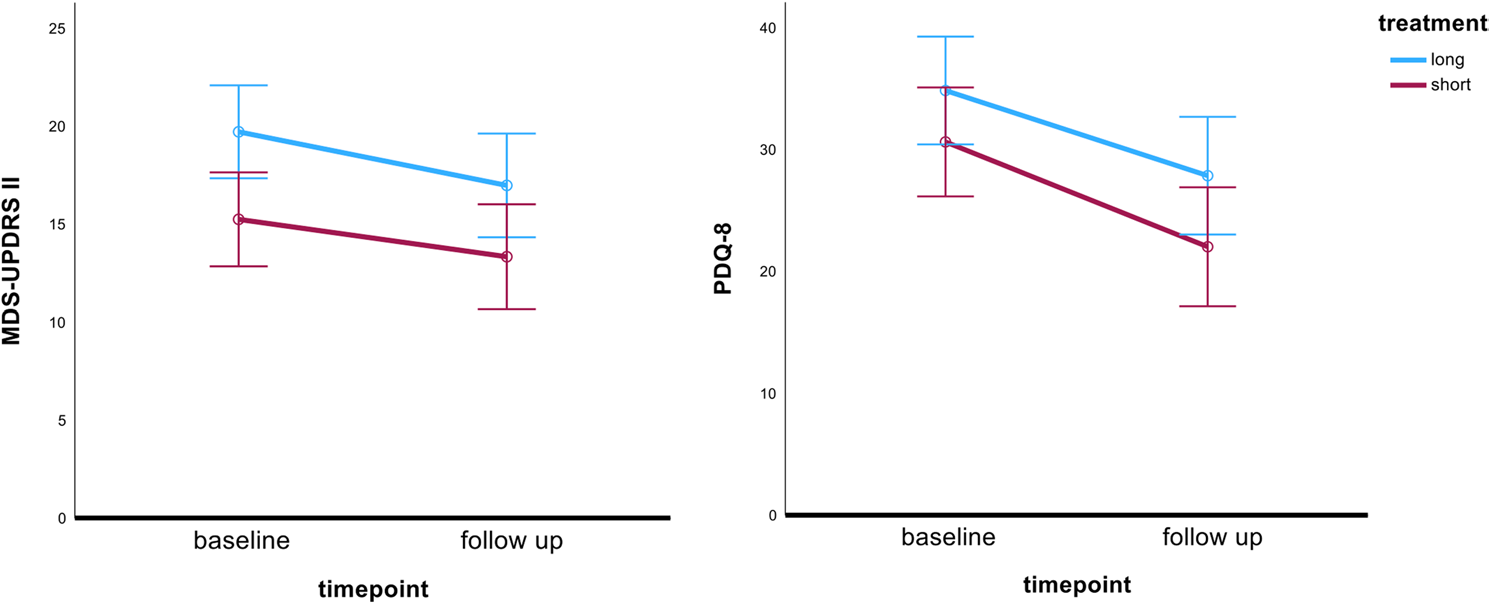
Mixed ANOVA. Improvements of the MDS-UPDRS II and PDQ-8 from baseline to follow-up with the within-subject factor timepoint (baseline; follow up) and the between-subject factor treatment duration (short; long)

Lastly, we performed a mixed ANOVA (Fig. 2) with the within-subject factor timepoint (baseline/follow up) and the between-subject factor treatment duration (short/long) to assess the influence of the treatment duration on the MDS-UPDRS II and PDQ-8.

When looking at the MDS-UPDRS II, a main effect of timepoint emerged (F(1,89) = 8.23, *p* = 0.005, Eta²= 0.018) confirming that patients’ scores improved in the six months after hospital discharge (mean 15.2 ± 9.2 points) in comparison to baseline (mean 17.4 ± 8.3 points). Additionally, a small but significant effect of treatment duration emerged (F(1,89) = 6.37, *p* = 0.013, Eta²= 0.054) suggesting that PwPD in the long group generally had a higher score (mean 18.3 ± 9.1 points) than those in the short group (mean 14.3 ± 8.0 points). However, as this pattern remained the case at both timepoints, there was no interaction of treatment and time (F(1,89) = 0.26, *p* = 0.611, Eta²= 0.001). This is because at baseline, the long PT-MCT group already performed worse (mean 19.6 ± 8.2 points) than the short group (mean 15.2 ± 7.9 points); and while both groups improved in their MDS-UPDRS II scores, the longer treatment duration (mean 17.0 ± 9.9 points) was not able to outperform the shorter treatment (mean 13.3 ± 8.1 points).

Regarding PDQ-8, the ANOVA (Fig. 2) showed a significant main effect of timepoint (F(1,91) = 25.30, *p* < 0.001, Eta² = 0.063), meaning that PwPD overall had significantly worse HRQoL scores at baseline (mean 32.9 ± 15.5 points) than at follow-up (mean 24.8 ± 16.6 points). There was neither a main effect of treatment (F(1,91) = 3.81, *p* = 0.054, Eta²= 0.031) nor an interaction between treatment and timepoint (F(1,91) = 0.06, *p* = 0.809, Eta²= 0.001), indicating no difference in the improvement between the two treatment groups.

## Discussion

In Germany, PD-MCT is increasingly implemented in inpatient care as a treatment option for PwPD. However, evidence on its long-term effects and the influence of treatment duration remains limited. Therefore, we conducted a prospective clinical trial with baseline allocation, collecting longitudinal data from 93 PwPD six months after discharge.

Our findings demonstrate sustained improvements in activities of daily living (MDS-UPDRS II) and HRQoL (PDQ-8). Considering minimal clinically important differences [26, 28], more than half of the cohort showed a clinically relevant long-term response. Specifically, 52.7% of PwPD improved in MDS-UPDRS II and 58.1% in PDQ-8 after six months. These results clearly extend previous reports describing beneficial effects lasting four to six weeks [12–15].

The median improvement in MDS-UPDRS II was 3 points, which is comparable to previously reported mean improvements of 2.2 points and 2.4 points after a follow-up of four to six weeks [13, 15]. The use of the median reflects the non-normal distribution of longitudinal changes in our cohort. HRQoL improved by a mean of 8.1 ± 15.5 points in the PDQ-8, with significant improvements in the items related to the social support, bodily discomfort, communication, concentration, and stigma domains of the PDQ-39 questionnaire. These findings are consistent with previous studies, especially regarding social support and bodily discomfort [12].

Beyond overall treatment effects, it is of particular clinical importance to identify factors that predict long-term treatment response. Improvement in MDS-UPDRS II was associated with higher MDS-UPDRS II scores, lower Hoehn and Yahr stage, and fewer nonmotor symptoms at admission. Improvement in HRQoL was associated with more depressive symptoms and again a lower Hoehn and Yahr stage. However, the explained variance of HRQoL response was limited (Nagelkerke *R*^2^ = 0.15) [27]. The association between baseline MDS-UPDRS II and treatment response should be interpreted with caution, as baseline values are mathematically related to change scores and may partly reflect regression-to-the-mean effects rather than purely clinical predictors.

Consistent with previous findings, baseline disease severity, as measured by the MDS-UPDRS II score, appears to influence treatment response [15]. Notably, PwPD with lower Hoehn and Yahr stages had a higher likelihood of long-term benefit in both MDS-UPDRS II and HRQoL. This is supported by a previous study which found that lower LEDD was associated with improved MDS-UPDRS II after PD-MCT [13]. These findings are of special interest, as this treatment concept has so far been particularly recommended for people with advanced stages of PD [29]. However, stratification based solely on motor severity does not account for the complexity of the disease. In this context, one should mention the impact of various nonmotor symptoms, particularly on the patient’s HRQoL [6, 30]. This is underlined by our results, revealing an association of more depressive symptoms at admission and a long-term improvement of HRQoL after PD-MCT. Given prior evidence of improved depressive symptoms after PD-MCT [12], it is plausible that the multidisciplinary approach alleviates depressive symptoms, thereby contributing to sustained HRQoL improvements. However, this hypothesis requires confirmation, and future studies should clarify which specific therapeutic components of PD-MCT are most effective in improving somatic and affective aspects of depressive symptoms [31, 32].

The sustained effects observed in our study may be explained by the holistic approach of PD-MCT. Beyond motor symptom control, the treatment likely strengthens patient resources and self-management strategies, including medication management, physical activity, self-monitoring, psychological support, independence, social engagement, and information [33]. These effects may extend beyond hospital discharge. However, future studies are needed to clarify which strategies are most beneficial and if tailored interventions can be integrated into PD-MCT.

Regarding treatment duration, most PwPD in Germany receive PD-MCT for at least 14 days [9], and existing evidence has mainly focused on the longer treatment duration. Only one retrospective study described that the shorter treatment duration (7–13 days) is also effective [14]. In our study, treatment duration was not significantly associated with long-term outcomes. However, the study was conducted within a pragmatic clinical framework reflecting routine care conditions. Although patients were initially allocated to predefined treatment duration categories, treatment duration could be modified during hospitalization for clinical or ethical reasons. Consequently, the analyses were conducted as-treated. While this approach enhances external validity and reflects clinical routine, it reduces experimental control and limits causal interpretation, particularly with regard to differences in treatment duration.

Importantly, baseline differences between the short and long PD-MCT groups, particularly regarding disease severity, further restrict the ability to draw causal conclusions from comparisons of treatment durations. These differences likely reflect clinical decision-making and underscore the pragmatic nature of the study design. Nevertheless, our data suggest that shorter PD-MCT may be a feasible option for certain patients, especially those with less severe disease or early clinical improvement. In light of the increasing prevalence of PD, individualized treatment durations could improve resource allocation and access to this highly specialized treatment [34, 35]. However, prospective studies are required to define subgroups suitable for shorter interventions.

Despite the promising results, our study has some limitations. First, our study used monocentric data, which limits its generalizability. However, as the treatment is standardized within Germany, a certain degree of transferability can be assumed. In this context, it is important to note that, although PD-MCT is a German OPS-based treatment concept embedded in the national reimbursement system, the principle of a structured, multidisciplinary inpatient approach for PwPD is not unique to Germany. Similar multidisciplinary, rehabilitation-oriented care models have been described internationally [36]. Therefore, our findings may be transferable at a conceptual level to other countries. Nevertheless, differences in healthcare organization, reimbursement structures, and the availability of specialized multidisciplinary teams may hinder the direct implementation of PD-MCT in its current form in other countries. Second, although randomization was computer-based, allocation was not blinded, and analyses were conducted as-treated. We chose this approach to reflect real-world inpatient care. However, this may have introduced bias in treatment assignment and limits causal interpretation of differences between treatment-duration groups [37]. Third, several potential confounders were not systematically assessed. In particular, physical activity, disease duration, and motor subtype were not included in the predefined multivariable models, although these factors may influence both activities of daily living and HRQoL. Future studies should incorporate standardized assessments of these variables to better characterize predictors of treatment response. Moreover, one has to mention that considering baseline MDS-UPDRS II as a predictor of MDS-UPDRS II response is mathematically related. However, our aim was to identify predictors of significant improvement, rather than the extent of improvement. Fourth, outcomes were based exclusively on patient reported measures, as the follow-up was carried out by telephone. In addition, loss to follow-up and the inability to account for a relevant number of patients transitioning to residential care may have introduced bias in our results, as those PwPD who did not benefit from PD-MCT may have been excluded from follow-up. The absence of clinical follow-up examinations to better assess the severity of PD limits conclusions regarding disease progression. Finally, our study did not examine cost aspects. In particular, it does not include patient-level cost data, post-discharge healthcare utilization, or utility-based outcomes. Therefore, a robust estimate of the cost-effectiveness of PD-MCT cannot be derived. Future studies should integrate long-term clinical follow-up with formal health economic analyses.

## Conclusions

In conclusion, our study revealed that PD-MCT is an effective inpatient treatment approach with a clinically relevant long-term effect for up to six months regarding motor experiences of daily living and HRQoL. In clinical practice, a shorter treatment duration may be feasible for selected patients and achieve similar long-term outcomes, but causal inference is limited.

## Supplementary Information

Supplementary Material 1.

## Data Availability

The datasets used and/or analysed during the current study are available from the corresponding author on reasonable request.

## Abbreviations

HRQoL: Health-related quality of life
IQR: Interquartile range
LEDD: Levodopa equivalent daily dose
MCID: Minimal clinically important difference
MDS-UPDRS: Movement Disorder Society sponsored revision of the unified Parkinson’s disease rating scale
MoCA: Montreal Cognitive Assessment
n: Number of patients
NMSQ: Non-Motor Symptoms Questionnaire
OPS: Operation and Procedure Classification System
OR: Odds Ratio
PD: Parkinson´s disease
PD-MCT: Parkinson´s disease multimodal complex treatment
PDQ-8: Parkinson’s Disease Questionnaire-8
PHQ-9: Patient Health Questionnaire-9
PwPD: People with Parkinson’s disease
SD: Standard deviation

## References

[1] Titova N, Qamar MA, Chaudhuri KR. The Nonmotor Features of Parkinson’s Disease. Int Rev Neurobiol. 2017;132:33–54. 10.1016/bs.irn.2017.02.016. 28554413 10.1016/bs.irn.2017.02.016

[2] Fereshtehnejad S-M, Romenets SR, Anang JBM, Latreille V, Gagnon J-F, Postuma RB. New Clinical Subtypes of Parkinson Disease and Their Longitudinal Progression: A Prospective Cohort Comparison With Other Phenotypes. JAMA Neurol. 2015;72:863–73. 10.1001/jamaneurol.2015.0703. 26076039 10.1001/jamaneurol.2015.0703

[3] Fereshtehnejad S-M, Zeighami Y, Dagher A, Postuma RB. Clinical criteria for subtyping Parkinson’s disease: biomarkers and longitudinal progression. Brain. 2017;140:1959–76. 10.1093/brain/awx118. 28549077 10.1093/brain/awx118

[4] Martinez-Martin P, Rodriguez-Blazquez C, Kurtis MM, Chaudhuri KR, Group on B of the NV. The impact of non-motor symptoms on health-related quality of life of patients with Parkinson’s disease. Mov Disord. 2011;26:399–406. 10.1002/mds.23462. 21264941 10.1002/mds.23462

[5] García DS, Fonticoba T, de D, Castro ES, Borrué C, Mata M, Vila BS, et al. Non-motor symptoms burden, mood, and gait problems are the most significant factors contributing to a poor quality of life in non-demented Parkinson’s disease patients: Results from the COPPADIS Study Cohort. Parkinsonism Relat Disord. 2019;66:151–7. 10.1016/j.parkreldis.2019.07.031. 31409572 10.1016/j.parkreldis.2019.07.031

[6] Barone P, Erro R, Picillo M. Quality of Life and Nonmotor Symptoms in Parkinson’s Disease. Int Rev Neurobiol. 2017;133:499–516. 10.1016/bs.irn.2017.05.023. 28802930 10.1016/bs.irn.2017.05.023

[7] Armstrong MJ, Okun MS. Diagnosis and Treatment of Parkinson Disease: A Review. JAMA. 2020;323:548–60. 10.1001/jama.2019.22360. 32044947 10.1001/jama.2019.22360

[8] Bloem BR, Okun MS, Klein C. Parkinson’s disease. Lancet. 2021;397:2284–303. 10.1016/S0140-6736(21)00218-X. 33848468 10.1016/S0140-6736(21)00218-X

[9] Richter D, Bartig D, Muhlack S, Hartelt E, Scherbaum R, Katsanos AH, et al. Dynamics of Parkinson’s Disease Multimodal Complex Treatment in Germany from 2010–2016: Patient Characteristics, Access to Treatment, and Formation of Regional Centers. Cells. 2019;8:151. 10.3390/cells8020151. 30754730 10.3390/cells8020151 PMC6406830

[10] Statistisches Bundesamt (Destatis). Fallpauschalenbezogene Krankenhausstatistik (DRG-Statistik) Operationen und Prozeduren der vollstationären Patientinnen und Patienten in Krankenhäusern (4-Steller) – 2022. 2023.

[11] Müller T, Öhm G, Eilert K, Möhr K, Rotter S, Haas T, et al. Benefit on motor and non-motor behavior in a specialized unit for Parkinson’s disease. J Neural Transm. 2017;124:715–20. 10.1007/s00702-017-1701-3. 28247031 10.1007/s00702-017-1701-3

[12] Scherbaum R, Hartelt E, Kinkel M, Gold R, Muhlack S, Tönges L. Parkinson’s Disease Multimodal Complex Treatment improves motor symptoms, depression and quality of life. J Neurol. 2020;267:954–65. 10.1007/s00415-019-09657-7. 31797086 10.1007/s00415-019-09657-7

[13] Hartelt E, Scherbaum R, Kinkel M, Gold R, Muhlack S, Tönges L. Parkinson’s Disease Multimodal Complex Treatment (PD-MCT): Analysis of Therapeutic Effects and Predictors for Improvement. J Clin Med. 2020;9:1874. 10.3390/jcm9061874. 32560079 10.3390/jcm9061874 PMC7356837

[14] Heimrich KG, Prell T. Short- and Long-Term Effect of Parkinson’s Disease Multimodal Complex Treatment. Brain Sci. 2021;11:1460. 10.3390/brainsci11111460. 34827459 10.3390/brainsci11111460 PMC8615811

[15] Ziegler K, Messner M, Paulig M, Starrost K, Reuschenbach B, Fietzek UM, et al. Activities of Daily Living Are Improved by Inpatient Multimodal Complex Treatment for PD—a Real-World Cohort Study. Mov Disord Clin Pract. 2022;10:42–54. 10.1002/mdc3.13578. 36698998 10.1002/mdc3.13578 PMC9847313

[16] Postuma RB, Berg D, Stern M, Poewe W, Olanow CW, Oertel W, et al. MDS clinical diagnostic criteria for Parkinson’s disease. Mov Disord. 2015;30:1591–601. 10.1002/mds.26424. 26474316 10.1002/mds.26424

[17] Goetz CG, Tilley BC, Shaftman SR, Stebbins GT, Fahn S, Martinez-Martin P, et al. Movement Disorder Society-sponsored revision of the Unified Parkinson’s Disease Rating Scale (MDS-UPDRS): scale presentation and clinimetric testing results. Mov Disord. 2008;23:2129–70. 10.1002/mds.22340. 19025984 10.1002/mds.22340

[18] Jenkinson C, Fitzpatrick R, Peto V, Greenhall R, Hyman N. The PDQ-8: Development and validation of a short-form parkinson’s disease questionnaire. Psychol Health. 1997;12:805–14. 10.1080/08870449708406741.

[19] Jenkinson C, Fitzpatrick R, Peto V, Harris R, Saunders P. PDQ-39 user manual (including PDQ-8 and PDQ summary index). The parkinson’s disease questionnaire. 2008.

[20] Goetz CG, Poewe W, Rascol O, Sampaio C, Stebbins GT, Counsell C, et al. Movement Disorder Society Task Force report on the Hoehn and Yahr staging scale: status and recommendations. Mov Disord. 2004;19:1020–8. 10.1002/mds.20213. 15372591 10.1002/mds.20213

[21] Schade S, Mollenhauer B, Trenkwalder C. Levodopa Equivalent Dose Conversion Factors: An Updated Proposal Including Opicapone and Safinamide. Mov Disord Clin Pract. 2020;7:343–5. 10.1002/mdc3.12921. 32258239 10.1002/mdc3.12921 PMC7111582

[22] Romenets SR, Wolfson C, Galatas C, Pelletier A, Altman R, Wadup L, et al. Validation of the non-motor symptoms questionnaire (NMS-Quest). Parkinsonism Relat Disord. 2012;18:54–8. 10.1016/j.parkreldis.2011.08.013. 21917501 10.1016/j.parkreldis.2011.08.013

[23] Nasreddine ZS, Phillips NA, Bédirian V, Charbonneau S, Whitehead V, Collin I, et al. The Montreal Cognitive Assessment, MoCA: a brief screening tool for mild cognitive impairment. J Am Geriatr Soc. 2005;53:695–9. 10.1111/j.1532-5415.2005.53221.x. 15817019 10.1111/j.1532-5415.2005.53221.x

[24] Kroenke K, Spitzer RL, Williams JBW. The PHQ-9. J Gen Intern Med. 2001;16:606–13. 10.1046/j.1525-1497.2001.016009606.x. 11556941 10.1046/j.1525-1497.2001.016009606.x PMC1495268

[25] Tinetti ME. Performance-oriented assessment of mobility problems in elderly patients. J Am Geriatr Soc. 1986;34:119–26. 10.1111/j.1532-5415.1986.tb05480.x. 3944402 10.1111/j.1532-5415.1986.tb05480.x

[26] Horváth K, Aschermann Z, Kovács M, Makkos A, Harmat M, Janszky J, et al. Minimal clinically important differences for the experiences of daily living parts of movement disorder society-sponsored unified Parkinson’s disease rating scale. Mov Disord. 2017;32:789–93. 10.1002/mds.26960. 28218413 10.1002/mds.26960

[27] Cohen J. Statistical Power Analysis for the Behavioral Sciences. 2nd edition. New York: Routledge; 1988. 10.4324/9780203771587.

[28] Luo N, Tan LCS, Zhao Y, Lau P-N, Au W-L, Li SC. Determination of the longitudinal validity and minimally important difference of the 8-item Parkinson’s Disease Questionnaire (PDQ-8). Mov Disord. 2009;24:183–7. 10.1002/mds.22240. 18972545 10.1002/mds.22240

[29] Krüger R, Klucken J, Weiss D, Tönges L, Kolber P, Unterecker S, et al. Classification of advanced stages of Parkinson’s disease: translation into stratified treatments. J Neural Transm (Vienna). 2017;124:1015–27. 10.1007/s00702-017-1707-x. 28342083 10.1007/s00702-017-1707-x PMC5514193

[30] Duncan GW, Khoo TK, Yarnall AJ, O’Brien JT, Coleman SY, Brooks DJ, et al. Health-related quality of life in early Parkinson’s disease: the impact of nonmotor symptoms. Mov Disord. 2014;29:195–202. 10.1002/mds.25664. 24123307 10.1002/mds.25664

[31] Heimrich KG, Mendorf S, Schönenberg A, Santos-García D, Mir P, Coppadis Study Group null. Depressive Symptoms and Their Impact on Quality of Life in Parkinson’s Disease: An Exploratory Network Analysis Approach. J Clin Med. 2023;12:4616. 10.3390/jcm12144616. 37510732 10.3390/jcm12144616 PMC10380984

[32] Stohlman SL, Barrett MJ, Sperling SA. Factor structure of the BDI-II in Parkinson’s disease. Neuropsychology. 2021;35:540–6. 10.1037/neu0000739. 33914574 10.1037/neu0000739

[33] Tuijt R, Tan A, Armstrong M, Pigott J, Read J, Davies N, et al. Self-Management Components as Experienced by People with Parkinson’s Disease and Their Carers: A Systematic Review and Synthesis of the Qualitative Literature. Parkinsons Dis. 2020;2020:8857385. 10.1155/2020/8857385. 33489082 10.1155/2020/8857385 PMC7787805

[34] Dorsey ER, Sherer T, Okun MS, Bloem BR. The Emerging Evidence of the Parkinson Pandemic. J Parkinsons Dis 8 Suppl 1:S3–8. 10.3233/JPD-181474. 10.3233/JPD-181474 PMC6311367 30584159

[35] Shaibdat NS, Ahmad N, Azmin S, Ibrahim NM. Causes, factors, and complications associated with hospital admissions among patients with Parkinson’s disease. Front Neurol. 2023;14:1136858. 10.3389/fneur.2023.1136858. 36959822 10.3389/fneur.2023.1136858 PMC10027758

[36] Steendam-Oldekamp E, van Laar T. The Effectiveness of Inpatient Rehabilitation in Parkinson’s Disease: A Systematic Review of Recent Studies. J Parkinsons Dis. 2024;14:S93–112. 10.3233/JPD-230271. 38788087 10.3233/JPD-230271 PMC11380234

[37] Smith VA, Coffman CJ, Hudgens MG. Interpreting the Results of Intention-to-Treat, Per-Protocol, and As-Treated Analyses of Clinical Trials. JAMA. 2021;326:433–4. 10.1001/jama.2021.2825. 34342631 10.1001/jama.2021.2825 PMC8985703

